# TRPA1 Influences *Staphylococcus aureus* Skin Infection in Mice and Associates with HIF-1a and MAPK Pathway Modulation

**DOI:** 10.3390/ijms25189933

**Published:** 2024-09-14

**Authors:** Manoj Yadav, Prem Prashant Chaudhary, Grace Ratley, Brandon D’Souza, Mahaldeep Kaur, Sundar Ganesan, Juraj Kabat, Ian A. Myles

**Affiliations:** 1 Epithelial Therapeutic Unit, National Institute of Allergy, and Infectious Disease, National Institutes of Health, Bethesda, MD 20892, USA; premprashant.chaudhary@nih.gov (P.P.C.); grace.ratley@nih.gov (G.R.); dsouza.80@buckeyemail.osu.edu (B.D.); mahaldeep.kaur@nih.gov (M.K.); ian.myles@niaid.nih.gov (I.A.M.); 2 Biological Imaging Section, Research Technologies Branch, National Institute of Allergy and Infectious Diseases, National Institutes of Health, Bethesda, MD 20892, USA; sundar.ganesan@nih.gov (S.G.); juraj.kabat@niaid.nih.gov (J.K.)

**Keywords:** TRPV1, TRPA1, MRSA, *staphylococcus aureus*, metabolomics

## Abstract

Infections caused by methicillin-resistant *Staphylococcus aureus* (MRSA) are a major public health burden. Emerging antibiotic resistance has heightened the need for new treatment approaches for MRSA infection such as developing novel antimicrobial agents and enhancing the host’s defense response. The thermo-ion channels Transient Receptor Potential (TRP-) A1 and V1 have been identified as modulators of *S. aureus* quorum sensing in cell culture models. However, their effects on in vivo infection control are unknown. In this study, we investigated the therapeutic effect of natural TRP ion channel inhibitors on MRSA skin infection in mice. While deletion of TRPV1 did not affect lesion size or inflammatory markers, TRPA1^−/−^ mice demonstrated significantly reduced infection severity and abscess size. Treatment with natural inhibitors of TRPA1 with or without blockade of TRPV1 also reduced abscess size. Tissue transcriptomic data coupled with immunohistochemistry revealed that TRPA1 inhibition impacted heat shock protein expression (HSP), modulated the HIF-1a and MAPK pathways, and reduced IL4 expression. Additionally, metabolomics data showed an impact on purine and glycosaminoglycan pathways. Multi-omic integration of transcriptomic and metabolic data revealed that diacylglycerol metabolism was the likely bridge between metabolic and immunological impacts. Our findings suggest that TRPA1 antagonism could provide a promising and cost-effective therapeutic approach for reducing the severity of MRSA infection, and presents a novel underlying molecular mechanism.

## 1. Introduction

Due to increasing rates of antibiotic-resistant infections, methicillin-resistant *Staphylococcus aureus* (MRSA) has become a pressing public health burden worldwide [1]. Hospitals, health centers, and communities are at increased risk due to MRSA, a major cause of wounds and surgical infections [2], and may require development of novel therapeutic approaches [3]. The pain that accompanies infection due to *S. aureus* is often processed through nociceptors and secondary immune regulators. Nociceptors express various molecular sensors for noxious and harmful stimuli. Sensory neurons also play a crucial role in communicating immunologic information from the skin to the brain, facilitated by specific proteins and receptors. Among these are the TRP ion channels, particularly Transient receptor potential ankyrin 1 (TRPA1) and Transient receptor potential vanilloid 1 (TRPV1) [4,5]. Recently in another study, it has been shown that TRPV1+ neurons alter *S. aureus* skin infection by affecting macrophage polarization mediated through calcitonin gene-related peptide (CGRP) [6]. 

Recent research has shown that MRSA can trigger pain sensations through the release of toxins and quorum-sensing molecules like N-acyl homoserine lactones acting through TRPA1 and TRPV1 [7,8]. The TRP channel plays a critical role in the detection and spread of infections by sensing bacterial endotoxins. [9,10]. However, despite the modeled role of TRP receptors in infection and immune modulation, the potential in vivo impacts for MRSA infection have not been established. In this study, we used transcriptomics coupled with metabolomics and immunohistochemistry (IHC) to investigate the role of TRPV1 and TRPA1 in MRSA infection control. The use of natural inhibitors of TRPA1 and TRPV1 (Table 1) will be cost-effective and will provide affordable treatment. Our results show that TRPA1 deletion or inhibition reduces MRSA lesion size via effects on HIF-1α, and MAPK pathways Imaging Mass Spectrometry findings propose that diacylglycerol metabolism may serve as a potential conduit linking metabolic and immunologic effects. Taken together, our multi-omics approach elucidated the mechanism of TRPA1 activity and identified TRPA1 as a therapeutic target for MRSA infection control.

## 2. Results

### 2.1. TRPA1 Modulates MRSA Infection

**Figure 1:**
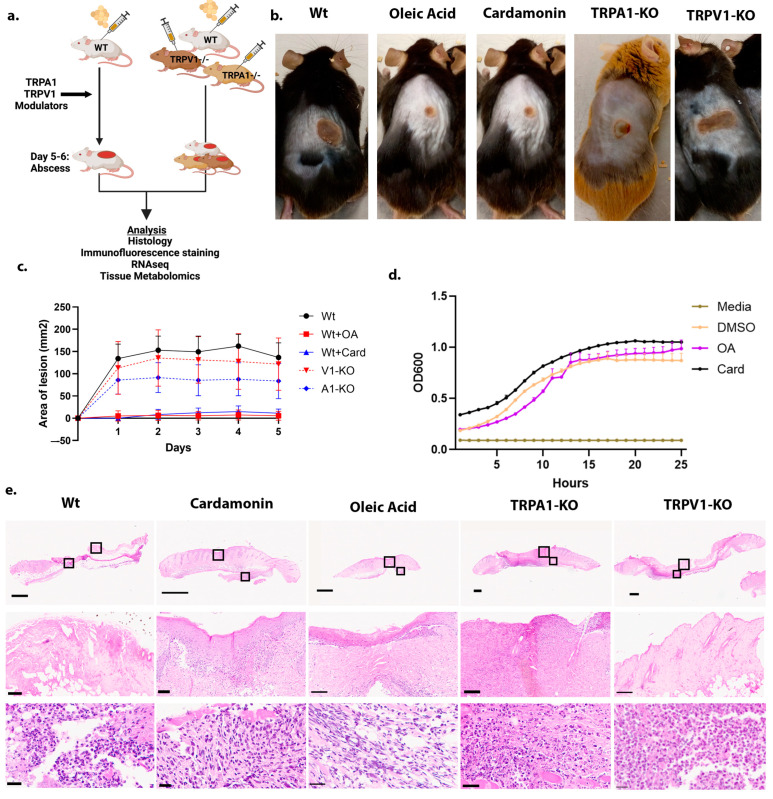
TRPA1-KO mice inhibit the MRSA infection. (**a**) Schematic representation of the experimental methodology. (**b**) Representative mice images after subcutaneous injection of MRSA and TRP modulators. Each mice treatment group had replicates *n* = 3–5 mice. Images shown are the mean of replicates from the group. (**c**) Quantification of lesion size from each treatment group. Multiple unpaired *t*-tests were performed for each data point to estimate significance. Statistical differences and *p*-value for each day data point are as follows: for (Wt + OA 0.000031, 0.000011, 0.000022, 0.000002, 0.000028) (Wt + Card 0.002609, 0.001869, 0.003648, 0.000768, 0.004007) (V1-KO 0.503670, 0.597096, 0.533226, 0.289300, 0.635908) (A1-KO 0.043400, 0.018122, 0.020217, 0.006397, 0.050155). (**d**) *S. aureus* growth curve after treatment with oleic acid (OA), cardamonin (Card), or DMSO for 24 h. (**e**) H and E stained images of skin lesions. H and E images show scale bars 1 mm, 100 µm, and 25 µm from low to high magnification. Black box shows enlarged images. Images were collected with the Hamamatsu Nanozoomer S60 scanner using a 40× objective.

Consistent with prior reports of TRPA1 modulation by lipopolysaccharide (LPS) and Staphylococcal quorum sensing compounds [5,8], subcutaneous injection of Methicillin-resistant *Staphylococcus aureus* (MRSA) strain USA-300 generated significantly smaller lesion size in TRPA1^−/−^ compared with wild-type mice (Figure 1a–c). In contrast, TRPV1 ^−/−^ mice presented with similar lesion sizes as wild-type mice. (Figure 1b). Subcutaneous injection of TRPA1 antagonist cardamonin, or dual blockade of TRPA1 and TRPV1 using oleic acid (OA), significantly reduced lesion size. To test whether cardamonin or oleic acid may affect *S. aureus* growth, we measured the optical density (OD) of the culture with or without treatment at 600 nm for 24 h. These inhibitors did not show an inhibitory effect on the growth of *S. aureus* (Figure 1d). Detailed quantification of H and E images did not show significant differences in neutrophil count compared to controls (Figure 1e). 

To evaluate previous reports of Th2-mediated potentiation of TRP channels [11], we assessed the presence of several markers of inflammation in infected skin tissue. IL-17A was reduced in TRPV1^−/−^ mice, while it was unaltered in TRPA1^−/−^ tissue (Supplementary Figure S1a,b). IL-20 was increased in both TRPA1^−/−^, and TRPV1^−/−^ tissue (Supplementary Figure S1a,c). IL-4 and the psoriasin signal were significantly reduced in both TRPA1^−/−^, and TRPV1^−/−^ mice (Supplementary Figure S1d–f).

### 2.2. HIF-1a and MAPK Pathways Are Altered in the Transcriptomes of MRSA-Infected TRPA1^−/−^ Mice

**Figure 2:**
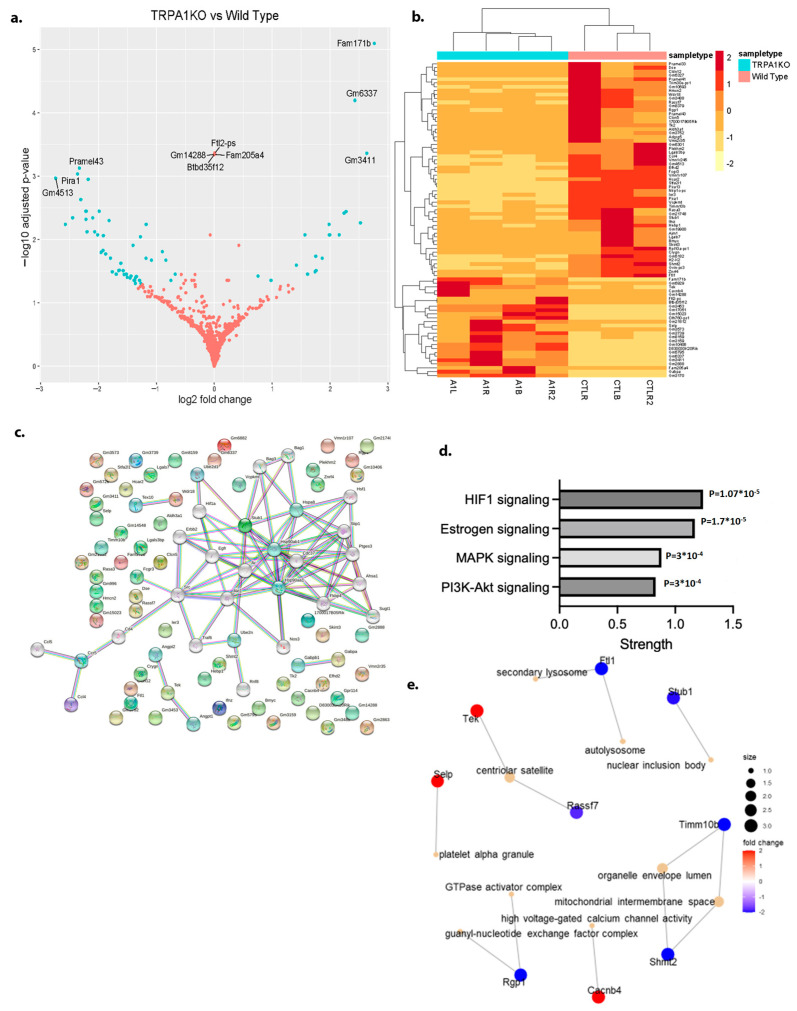
**Transcriptomics of TRPA1**^−/−^ **mice tissue**. (**a**) Volcano plot showing the differentially expressed genes (DEGs). Red dots represent non-significant genes, while green dots indicate significantly upregulated (fold change > 1.5) or downregulated (fold change < −1.5) genes. Threshold values for fold change are set at ±1.5. (**b**) Heatmap displaying the patterns of differentially expressed genes (DEGs) across all sample types, highlighting sample-to-sample variations in each group. (**c**) String analysis from the DEGs showing the important gene cluster in MRSA infection. (**d**) Summary of pathways from DEGs impacted in TRPA1^−/−^ mice. (**e**) CNET plot of relationships between significantly differentially expressed genes and their associated biological categories in MRSA infection.

Bulk RNA-seq on MRSA-infected skin of TRPA1^−/−^ mice revealed 82 genes that were differentially expressed (DEGs) (Figure 2a,b). TRP ion channels are key to maintaining internal temperature, homeostasis, and protecting skin from various environmental stressors through heat-shock proteins (HSPs) [12]. Pathway string analysis indicated [13] that the HIF-1 and MAPK signaling pathways were the most significantly impacted immunologic pathways centering on HSP90ab1 (Figure 2c,d). TRPA1^−/−^ and its antagonist increased expression of HIF-1a, a transcription factor that promotes angiogenesis and erythropoiesis under conditions of hypoxia and in response to oxidative stress [14] (Supplementary Figure S2a). To verify the expression of HIF1α, we used an anti-HIF1α antibody and performed IHC staining on MRSA-infected mice skin tissue sections. IHC staining showed that TRPA1^−/−^ and TRPV1^−/−^ mice presented with increased HIF1α expression compared to the control mice (Supplementary Figure S2b,c). Thus, the results obtained are in concordance with the RNAseq data. Downregulation of TRPA1^−/−^ affected other processes, such as secondary lysosome, mitochondrial intermembrane space, high voltage-gated Ca^2+^ channel activity, and guanyl nucleotide exchange factor complex (Figure 2e). Transcriptomics from TRPV1^−/−^ mice revealed only three significant DEGs (Supplemental Figure S3a,b).

### 2.3. TRPA1 Modulates Metabolic Response to MRSA Infection 

**Figure 3:**
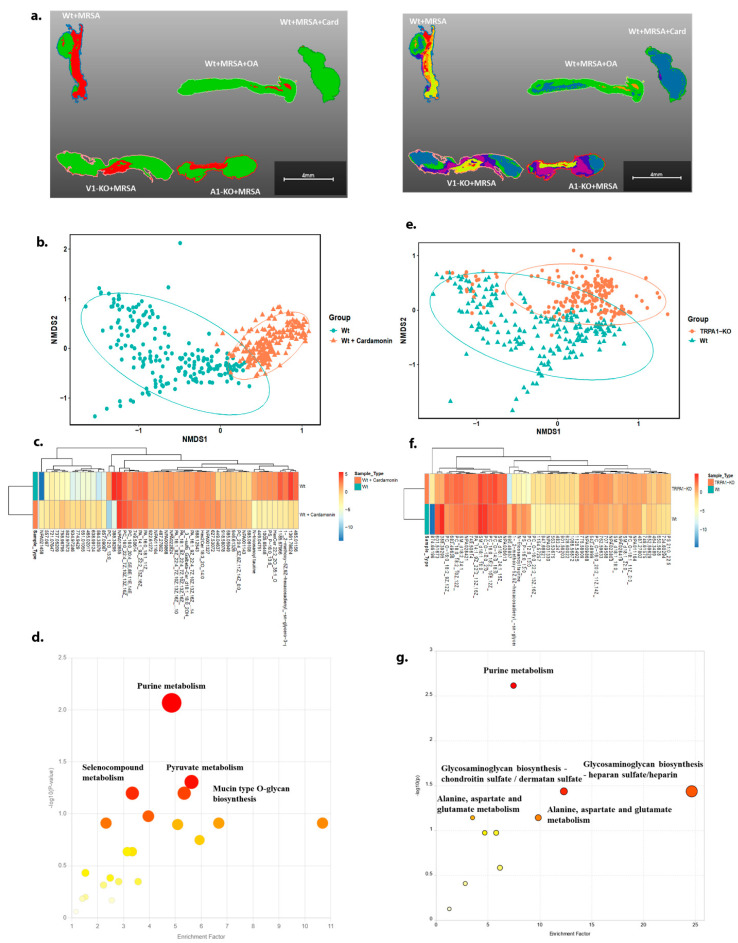
Metabolomics of TRPA1^−/−^ mice and cardamonin-treated mice. (**a**) Spatial distribution of metabolites from skin tissue isolated from TRPV1^−/−^ and TRPA1^−/−^ mice by MALDI-TOF imaging. Each color represents the presence of different metabolites in the tissue sample. (**b**) NMDS plot illustrates the differences in the overall metabolite profiles between wild-type and cardamonin-treated mice skin tissue. The plot provides a visual representation of the dissimilarities in metabolite composition, highlighting how the treatment with cardamonin affects the metabolic state of the skin tissue compared to the wild type. (**c**) The heat map plot shows the top 50 significant metabolites in wild-type vs. cardamonin-treated mice. (**d**) Shows significantly impacted pathways hit by MetaboAnalyst in cardamonin-treated mice. (**e**) NMDS plot shows the difference in the total metabolites in wild type and TRPA1^−/−^ mice skin tissue. (**f**) Heat map plot shows the top 50 significant metabolites in wild type vs. TRPA1^−/−^ mice. (**g**) Shows significantly impacted pathways hit by MetaboAnalyst in TRPA1^−/−^ mice. Color represents the level of significance.

Using MALDI imaging mass spectrometry, we were able to visualize the variations in metabolite distribution between the TRPA1^−/−^ mice and the wild-type (Wt) tissue (Figure 3a). Global metabolic differences were seen by Non-Metric Multi-dimensional Scaling (NMDS) segmentation (Figure 3b,e) as well as for specific metabolites identified by a combination of *m*/*z* and collisional cross section (CCS) (Figure 3c,f). Pathways analysis from MetaboAnalyst demonstrated that both purine metabolism and KEGG glycosaminoglycan pathways were significantly impacted in both TRPA1^−/−^ and cardamonin-treated mice (Figure 3d,g). Differentially expressed metabolites were imported in Pathview, which provides the pathways that were impacted upon treatment or in KO mice (Supplementary Figure S4a,b).

### 2.4. TRPV1 Modulates Metabolic Response to MRSA Infection 

Despite the lack of modeled clinical improvement, TRPV1^−/−^ and oleic acid-treated mice demonstrated global differences in metabolites as well as differences in specifically identified compounds (Supplementary Figure S5a–e). Pathway analysis using MetaboAnalyst revealed that upon treatment with OA, sphingolipid metabolism, drug metabolism, and selenocompound metabolism were modified (Supplementary Figure S5c). On the other hand, TRPV1^−/−^ mice had significant differences in glycosaminoglycan biosynthesis and glycosphingolipid biosynthesis pathways (Supplementary Figures S5f and S6a,b).

### 2.5. Multi-Omic Integration of TRPA1 Modulation Reveals DAG and Ceramides 

**Figure 4:**
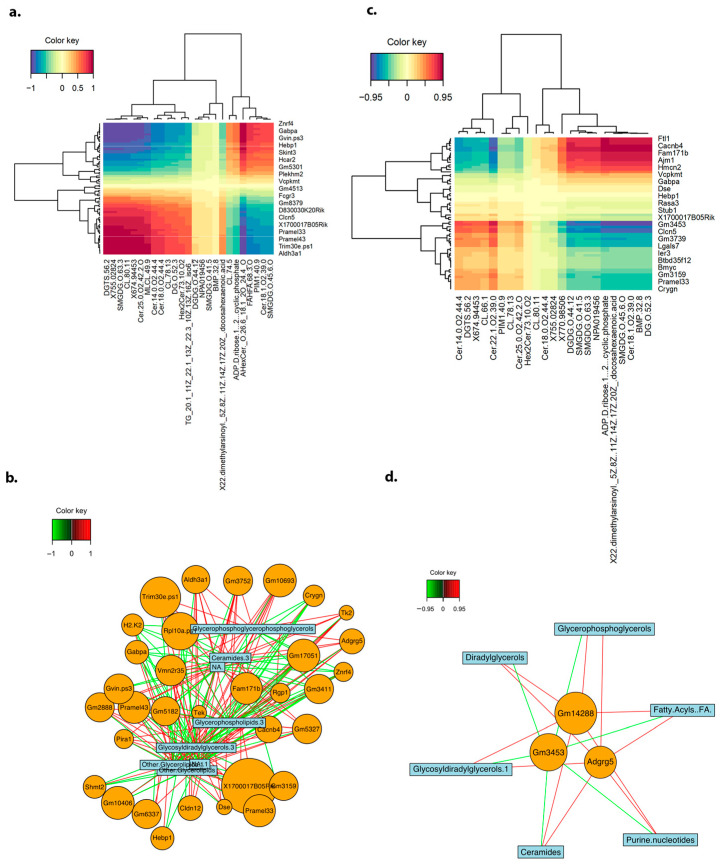
**Shows integration of transcriptomics and metabolomics data from wild-type mice and TRPA1^−/−^ mice.** (**a**) An integrated heatmap was created using significant DEGs from transcriptomics data and the most significantly different metabolites from the metabolomics data. It shows the strength and direction of relationships between metabolites and gene expressions, helping identify significant associations between them. In the wild-type mice group, this shows the significantly upregulated or downregulated genes and metabolites. (**b**) Network analysis from transcriptomics and metabolomics reveals how these significant DEG gene metabolites are associated with each other in wild-type mice. Red and green lines show positive and negative correlations in wild-type mice, respectively. (**c**) Similarly, an integrated heatmap was created by using significant DEGs from transcriptomics data and the most significantly different metabolites from the metabolomics data from TRPA1^−/−^ mice. (**d**) Network analysis from the transcriptomics and metabolomics reveals how these significant DEG gene metabolites are associated with each other in TRPA1^−/−^ mice. Red and green lines show positive and negative correlations respectively in TRPA1^−/−^ mice.

**Figure 5:**
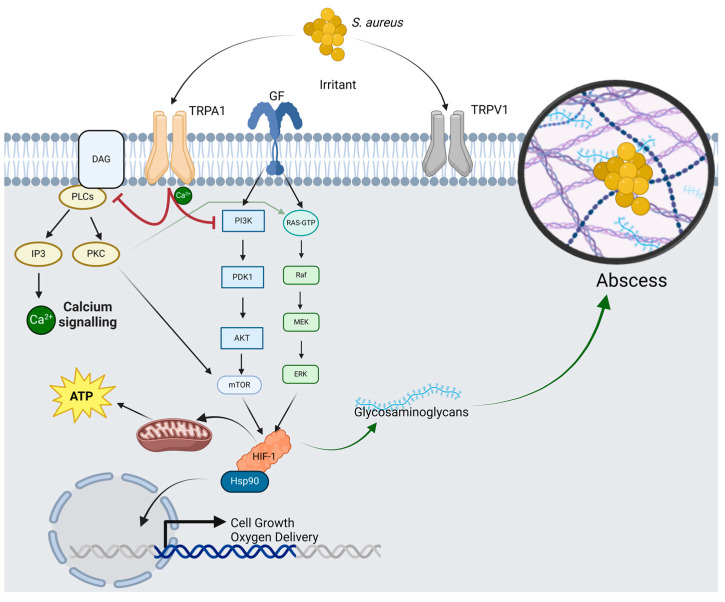
Shows signaling cascade and involvement of different molecules. Effect of TRPA1 and other downstream molecules such as HSP90 and HIF-1α in *S. aureus* infection. Abbreviations are as follows: Transient receptor potential ankyrin 1 (TRPA1), Diacylglycerol (DAG), Phospholipase C (PLC), Inositol tryphosphate (IP3), Protein kinase C (PKC), Adenosine try phosphate (ATP), Phosphoinositide 3-kinases (PI3K), 3-phosphoinositide-dependent kinase 1 (PDK1), Protein kinase B (AKT), Mammalian target of rapamycin (mTOR), Rat sarcoma guanosine triphosphatases (RAS-GTP), Rapidly Accelerated Fibrosarcoma (RAF), Mitogen-activated protein kinase (MEK), Extracellular signal-Regulated Kinase (ERK), Hypoxia-inducible factor (HIF-1), Heat shock protein 90 (HSP-90), Transient receptor potential vanilloid 1 (TRPV1).

We used the integration of transcriptomic and metabolomic data to understand signaling mechanisms and pathways affected by TRP modulation. Integrative heatmaps of genes and metabolites that were differentially expressed in wild-type and TRPA1^−/−^ mice illustrated the correlation or association between the expression levels of specific genes and the abundance of corresponding metabolites (Figure 4a,d). Omics integration of wild-type samples revealed that glycerophospholipid, ceramides, and other glycerophospholipids were clustered along with DEGs (Figure 4b). Integration of TRPA1^−/−^ mice data revealed that diacylglycerol (DAG) and phospholipids were clustered with the DEGs identified from the transcriptomic analyses (Figure 4d; Supplementary Table S1). A TRPA1^−/−^ network plot showed an interconnection between different subclasses of diacyl glycerol such as glycosyldiradylglycerols along with ceramides, fatty acyls, purine nucleotide, and sphingolipids (Figure 4d). Overall, TRPA1 activation in the context of MRSA infection correlated with established immune signaling pathways (Figure 5) [15,16,17,18]. This section may be divided by subheadings. It should provide a concise and precise description of the experimental results and their interpretation, as well as the experimental conclusions that can be drawn.

## 3. Discussion

While in vitro data has previously indicated an interaction between cutaneous TRP receptors and bacterial pathogens, there has been a lack of direct evidence demonstrating the modulation of infection control by TRPs. This study identified TRPA1 as a promising therapeutic target for MRSA infection. Both the genetic deletion of TRPA1 and the use of natural inhibitors yielded improved outcomes in MRSA-infected mice. Notably, our investigation suggested that TRPV1 may not play a direct role in MRSA control, as knockout mice did not exhibit enhanced responses. Moreover, the TRPA1/V1 blocker OA did not confer additional benefits beyond the inhibition of TRPA1 alone. 

Our transcriptomic data indicated that differential expression of known pathways of inflammation and infection response, such as HIF1α, MAPK, and PI3K signaling, may be impacted by TRP modulation. Multi-omic assessments helped fill gaps in understanding the links between metabolic and immunologic activities of TRP activation by implicating diacylglycerol metabolism as the potential link. Our IHC data further confirmed previously shown interactions between Th2 signaling and TRP modulation [11,18]. Immunohistochemistry revealed altered levels of IL20, IL17A, IL4, and psoriasin, which are involved in infection. Under homeostatic conditions, TRP channels are crucial in maintaining body temperature in different environmental and physical conditions through the modulated expression of heat shock proteins (HSP) [12,19]. This study further highlights the importance of HSP and TRP signaling, while adding insights into the connection between HSP like HSP90ab1 and HSP90aa1 and subsequent signaling through HIF1α and MAPK inflammatory pathways. Previous studies have shown that HIF-1α is upregulated in *S. aureus*-infected MC3T3-E1 cells and osteomyelitis patients [14]. Our findings further elucidate the potential mechanisms of the established connection between HIF1α and DAG [20,21,22] by suggesting that one consequence of this pathway includes modulations of the glycosaminoglycans, which are important precursors of abscess formation [23,24]. 

*S. aureus* is a known activator of PI3K and the RAS/ERK pathways [25], each of which induces HIF1α [26]. HIF1α, through interactions with HSP, induces cell growth and oxygen metabolism [27], which influences the production of the types of glycosaminoglycans [28] known to aid in abscess formation [23,24]. DAG interacts with this process by altering Ca^2+^ signaling through PLC, IP3, and PKC [15,16]. Our data suggest that TRPA1 influences both DAG and PI3K, resulting in the expected reduction of HIF1α activation, alteration in glycosaminoglycan metabolism, and ultimately worsened MRSA control due to altered abscess formation. Overall, we proposed that the established calcium signaling pathways of DAG and TRPA1 interact under immunometabolism. However, even while consistent with established signaling pathways, our mechanistic data at this time are limited to observations. Whether DAG or HSP are essential for TRPA1 modulation of MRSA infection remains to be elucidated. 

Our results are also limited in the use of only a single MRSA isolate (USA300) in mice; although it is the predominant MRSA isolate in the USA [29], we acknowledge that strain-level variations in *S. aureus* likely influence host response. Our results are consistent with the reported role of glycosaminoglycans in infection control and abscess formation [24,30,31]. The use of varied HSP inhibitors may further elucidate the mechanisms of TRPA1 modulation on MRSA control. In addition, subsequent work should evaluate the potential role of TRPV3, which is also expressed in the skin, but for which knockout mice are less available [32,33]. Newer data suggest the possible beneficial role of TRPA1 in the control of parasitic infections [34,35]; thus, further studies should compare if the processes that are deleterious for MRSA control may be of evolutionary benefit in helminth infection.

To our knowledge, this is the first in vivo study that directly assessed the clinical and signaling impact of the TRP receptors on MRSA infection and partially elucidated the immune-modulating activities of TRPA1. Overall, our findings suggest that TRPA1 may be a previously unrecognized target for improving the control of MRSA skin infections and elucidating some of the molecular mechanisms of TRPA1 modulation.

## 4. Materials and Methods

### 4.1. Reagents

Oleic Acid (# 75090-5ML, Sigma Aldrich, Saint Louis, MO, USA), Cardamonin (# C8249-5MG, Sigma aldrich, Saint Louis, MO, USA), DAPI (#62248, Life Technology, Carlsbad, CA, USA), mouse Alexa Fluor-488 (# A11001, Life Technology, Carlsbad, CA, USA), mouse Alexa Fluor-568 (# A11031, Life Technology, Carlsbad, CA, USA), rabbit Alexa Fluor 488 (# A11034, Life Technology, Carlsbad, CA, USA), rabbit Alexa Fluor 568 (# A11011, Life Technology, Carlsbad, CA, USA). Antibodies for IHC anti- Periostin (# 2955-F2-050, R and D systems, Minneapolis, MN, USA), IL-20 Monoclonal Antibody (OTI2B8) (# MA5-26604, Life Technology, Carlsbad, CA, USA)), Mouse IL-4 Antibody (# MAB404-100 R and D systems, Minneapolis, MN, USA), S100A7/Psoriasin Polyclonal Antibody (#PA5-96002, Life Technology, Carlsbad, CA, USA), IL-17F Polyclonal Antibody, Anti-TNF alpha antibody [52B83] (# ab1793, Abcam, Cambridge, UK), Anti-IL-1 beta antibody (# ab2105, Abcam), anti HIF1α antibody (NB100-134SS from Novas Biologicals, Centennial, CO, USA). 

### 4.2. Mice

All mice used here had a background of B6129PF2/J. Male and female mice Wt-JAX664, TRPA1-KO-JAX6401, and TRPV1-KO-JAX3770 aged 6–12 weeks were purchased from Jackson Labs (Bar Harbor, MA, USA). All experimental groups such as Wt, TRPA1^−/−^, and TRPV1^−/−^ have 5 mice at the beginning of the experiment. All mice were age- and sex-matched within each experiment. Lesional skin was collected and analyzed as described for the MC903 model [36]. For experiments using imaging mass spectrometry, mouse tissue was fresh-frozen and analyzed in positive ion mode, as previously described [37].

### 4.3. Bacterial Infection

*S. aureus* strain (USA300) was used for mice infection. The bacterial dose of 107 CFU was used for skin infection. For mice experiments, 500 and 250 µM concentrations of cardamonin and OA were used, respectively. One hundred microliters (100 µL) of *S. aureus* in PBS (107 CFU) was injected subcutaneously into the shaved back of mice. From the second day, the lesion was measured at the mid-point intersection for length and width using an electronic caliper. The area was calculated by multiplying the width and length of the lesion measured for six days.

### 4.4. Immunohistochemistry (IHC) and Immunocytochemistry

Mouse skins was formalin-fixed and was sectioned (10–20 µm) (Histoserv Inc., Germantown, MD, USA). Sectioned slides were immersed twice in xylene solution for 10 min each and 100% ethanol two times for 10 min each. Next, slides were sequentially immersed in 95%, 70%, and 50% ethanol for 5 min then washed with deionized H_2_O and soaked in PBS buffer for 10 min. Heat-induced antigen retrieval was performed on the tissue using epitope retrieval solution, pH 6 (# RE7113-CE, Leica, Deerfield, IL, USA), and an Instant Pot pressure cooker was set on “soup” at high pressure for 40 min. The tissue was surrounded by a hydrophobic barrier pen. Tissue sections were blocked with 5% Normal Goat Serum (NGS) for 1 h. The primary antibody solution was added according to the manufacturer's instructions at 4 °C overnight. *Slides* were washed thrice for 15 min each in a wash buffer. A secondary antibody solution was added according to the manufacturer's instructions for 1 h, followed by washing thrice for 15 min with the wash buffer. DAPI solution was added and incubated for 15 min at room temperature. Slides were washed 3 times with PBS and were mounted with a mounting medium.

Immunocytochemistry protocols were followed as previously described [37]. Cells were fixed with 4% PFA for 15 min at 37 °C, without disturbing the cells. Fixed solution was removed from the cells and cells were washed with 1× PBS three times followed by the addition of 0.5% TritonX 100 for 20 min. Cells were washed with 1× PBS. An amount of 5% NGS was added to the cells for 1 h. Primary antibody solution was added for 1 h and cells were washed 3 times with 1× PBS with 5 min incubation. The secondary antibody solution was added for 30 min. Cells were washed 3 times with 1× PBS with 5 min incubation. DAPI solution was added in 1× PBS for 20 min. Cells were washed with 1× PBS 3 times for 5 min. The glass coverslip was mounted with the mounting reagent. After 24 h, slides were imaged with a confocal microscope.

### 4.5. H and E Imaging

H- and E-stained tissue slides were imaged with a Hamamatsu Nanozoomer S60 scanner using a 40× objective. Images were visualized, processed, and quantified with NDP.view 2.9.29 software.

### 4.6. MALDI Tissue Imaging

Fresh frozen tissue collection, matrix spraying, data acquisition, and MALDI imaging were performed as described previously [37]. Briefly, 20 mg/mL of 5-dihydroxybenzoic acid [(#149357-20G, Sigma-Aldrich, Saint Louis, MO, USA; in 100% acetone and 0.1% trifluoroacetic acid (#302031, Sigma)] was used as the matrix solution. The tissues were scanned with both MS and TIMS settings at a resolution of 20 µm. The MS settings were scan range 20–2500 *m*/*z* in positive MS scan mode. The TIMS settings were: 1/K0 0–8 − 1.89 V × s/cm^2^, ramp time of 200 ms, acquisition time of 20 ms, duty cycle = −10%, and ramp rate of 4.85 Hz. Acquired raw data were initially processed with SCiLS lab 2021a (Bruker Scientific LLC, Billerica, MA, USA), and the file was exported to Metaboscape 2021b (Bruker, Billerica, MA, USA) for annotations and further downstream analysis. 

### 4.7. Metabolomic Analysis

Feature tables were exported from MetaboScape, and unpaired *t*-tests conducted in R were used to determine between-group differences in metabolite abundance. Non-metric dimensional scaling (NMDS) was carried out using the vegan package. To measure how groups differ based on the levels of all the metabolites found in the group, ANOSIM was employed [37]. Peak intensity data were log-transformed, and normalization was performed by mean subtraction. Unpaired *t*-tests were used to determine significantly different metabolites between the two groups. Metabolites sorted by FDR *p*-value were exported to MetaboAnalyst for functional analysis with the following parameters: ion mode was positive, mass tolerance was 5, and the mummichog algorithm was used to compare input data to the human KEGG database. From the enrichment data, the index of pathway significance (IPS) was calculated for each identified pathway. Log-transformed IPS values were visualized using iPath 3 [38].

### 4.8. RNA Isolation from Mice Skin

Mice skins were stored in RNAlater solution (#AM7021, Thermo Fisher Scientific, Waltham, MA, USA) at −20 °C. RNeasy Mini Kit (50) Cat. No. 74104 from Qiagen, Germantown, MD, USA Based on the manufacturer's instructions, skin tissue was added with 10 μL β-mercaptoethanol (β-ME) to 1 mL Buffer RLT, followed by adding 4 volumes of ethanol 100% to Buffer RPE for a working solution. The tissue was removed using forceps from RNAlater solutions. After weighing tissue not more than 30 mg, disrupt the tissue and homogenize the lysate in the appropriate volume of Buffer RLT. Centrifuge the lysate for 3 min at maximum speed. Carefully remove the supernatant by pipetting and use it in the next step. Add 1 volume of 70% ethanol to the lysate and mix well by pipetting. Transfer up to 700 μL of the sample, including any precipitate, to an RNeasy Mini spin column placed in a 2 mL collection tube and centrifuge for 15 s at ≥8000× *g*. Discard the flow-through. Add 700 μL Buffer RW1 to the RNeasy spin column, and centrifuge for 15 s at ≥8000× *g*. Discard the flow-through. Add 500 μL Buffer RPE to the RNeasy spin column, and centrifuge for 15 s at ≥8000× *g*. Discard the flow-through. Add 500 μL Buffer RPE to the RNeasy spin column, and centrifuge for 2 min at ≥8000× *g*. Place the RNeasy spin column in a new 2 mL collection tube. Centrifuge at full speed for 1 min to dry the membrane. Place the RNeasy spin column in a new 1.5 mL collection tube. Add 30 μL RNase-free water directly to the spin column membrane, and centrifuge for 1 min at ≥8000× *g* to elute the RNA. After extraction of RNA from tissue, the concentration of RNA was measured using a nanodrop, followed by cDNA synthesis (#1, ,2012801, Reliance Select cDNA Synthesis Kit, Hercules, CA, USA) according to the manufacturer's instructions. cDNA was used to prepare the double-stranded cDNA (# 11917-020, SuperScript Double-stranded cDNA synthesis kit Invitrogen, Waltham, MA, USA) according to the manufacturer’s instructions. Followed by RNA sequencing at CosmosID, Gaithersburg, MD, USA. 

### 4.9. RNA Seq Analysis and Visualization

Raw reads were filtered, and quality trimmed by using Trimmomatic software version 0.39–1 [39]. The Salmon software version 0.13.2 [40] was used to analyze our RNA-seq data by mapping the sequenced reads to the mus musculus reference transcriptome from Ensembl (GRCm39) using the quasi-alignment model. Gene expression is represented in transcripts per million (TPM). We used the R Bioconductor package version 3.19 tximport to convert the TPM into non-normalized count estimates for performing DESeq2 analysis and to carry out abundance estimates from the transcript level to the gene level.

Differentially expressed genes (DEGs) were identified with DESeq2 version 1.24.0 in R [41]. The estimated number of reads obtained from Salmon was used to run the DESeq2 pipeline. This DESeq2 pipeline also performs the normalization based on per-sample sequencing depth and the presence of intra-sample variability. Further, data were fitted into a negative binomial generalized linear model (GLM) using the Wald statistic. Finally, obtained *p*-values were adjusted for multiple comparisons using the false discovery rate (FDR). Genes were considered significantly different with a cutoff FDR < 0.05. Enhanced Volcano R package, 3.19 was used to generate the volcano plot. Significantly different genes were visualized using a heatmap.

### 4.10. Integration of Transcriptomics and Metabolomics

To integrate the results of transcriptomics and metabolomics data to extract correlated information, or by highlighting commonalities between data sets, we used sPLS-DA implemented in the MixOmics R package 3.19 [42]. To reduce the dimensionality of the input data to a manageable size, only differential features between the comparisons from transcriptomics and metabolomic datasets were used, resulting in the identification of a highly correlated multi-omics signature panel [43]. Metabolites identified by mummichog in MetaboAnalyst were subset and limma regression was used to determine fold change. Gene and metabolite fold change data were uploaded to Pathview, and pathway selection was performed using generally applicable gene set enrichment (GAGE).

### 4.11. Statistics

All statistics were performed using PRISM (GraphPad), as indicated in the respective figure legends. One-way ANOVA (Dunnetts multiple comparisons test) was performed for multiple comparisons. The *p*-values are as follows *** < 0.001, ** < 0.01, * < 0.05. For Figure 1c, multiple unpaired *t*-tests were performed for each data point to check the significance. 

## Figures and Tables

**Table 1:** List of TRP modulators and their target ion channels:

TRP Modulator	Target TRP Channel
Cardamonin	Inhibits TRPA1
Oleic acid	Inhibits TRPV1; partial inhibition TRPA1

## Data Availability

RNA seq data have been submitted for accessibility under the BioProject ID PRJNA918729, locus_tag_prefix PDX24, and biosample_accession number SAMN32605717.

## Supplementary Materials

The following supporting information can be downloaded at: https://www.mdpi.com/article/10.3390/ijms25189933/s1.
