# Supplementary material for: TRPA1 Influences Staphylococcus aureus Skin Infection in Mice and Associates with HIF-1a and MAPK Pathway Modulation
Source: Int J Mol Sci. 2024 Sep 14;25(18):9933. doi: 10.3390/ijms25189933 (PMC11432213; doi:10.3390/ijms25189933)
Supplement: Supplementary file 1 [file ijms-25-09933-s001.zip › ijms-3163627-supplementary.pdf]

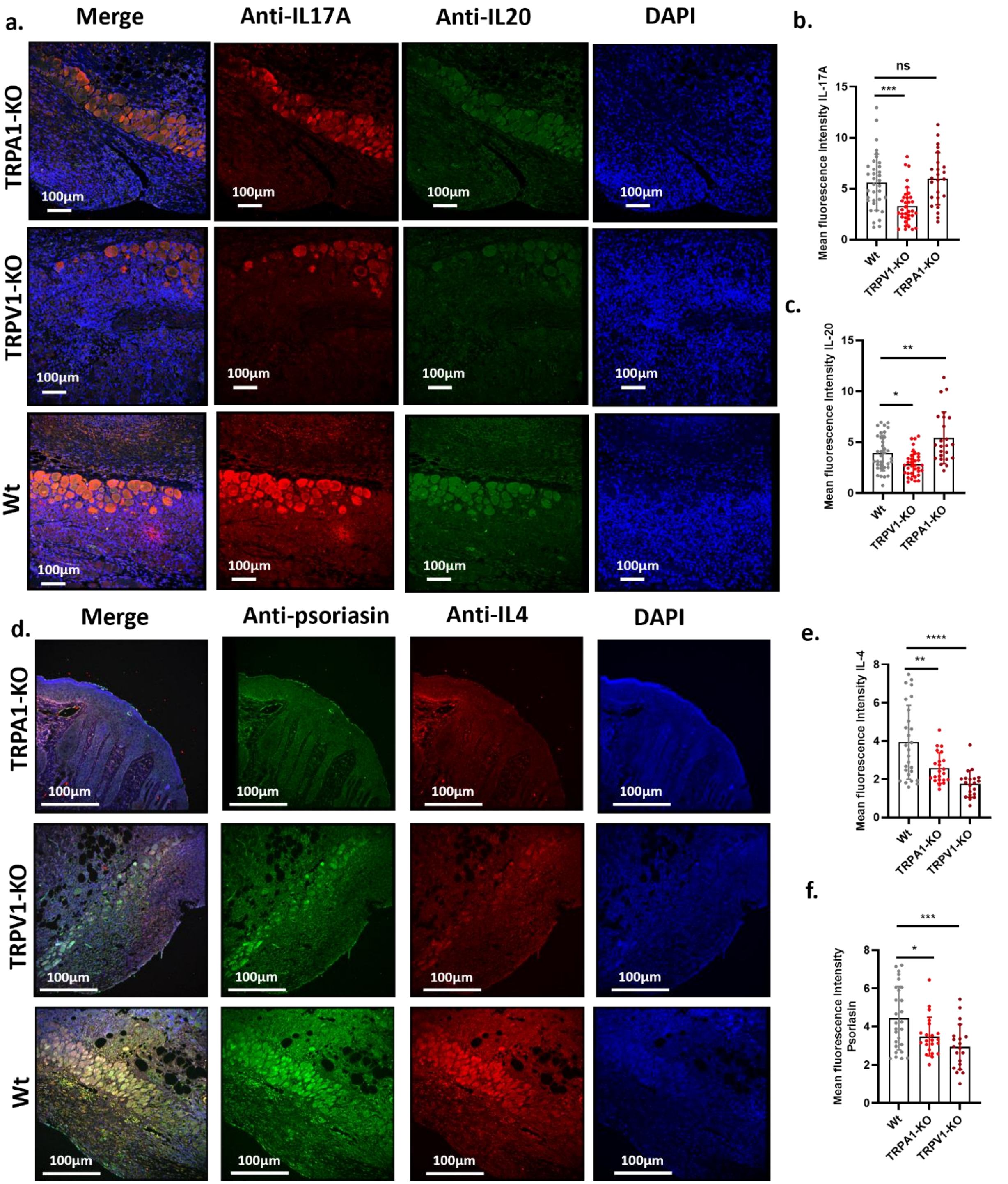

Supplementary Figure S1: Immunohistochemistry (IHC) of the skin tissue. (a). Confocal images show zoomed subcutaneous fat tissue. Images stained with IL17A, IL-20 and DAPI in each mice tissue group. **(b,c).** Quantification of Il17A and IL20 respectively. **(d).** Confocal images show zoomed epidermal and dermal tissue. Confocal images of Psoriasin, IL-4, and DAPI stained mice tissue. **(e,f).** Quantification of IL-4 and Psoriasin respectively. One way ANOVA (Dunnetts multiple comparisons test) was performed for multiple comparison. The *p*-values are as follows ****<0.0001,*** <0.001, ** <0.01, * <0.05.

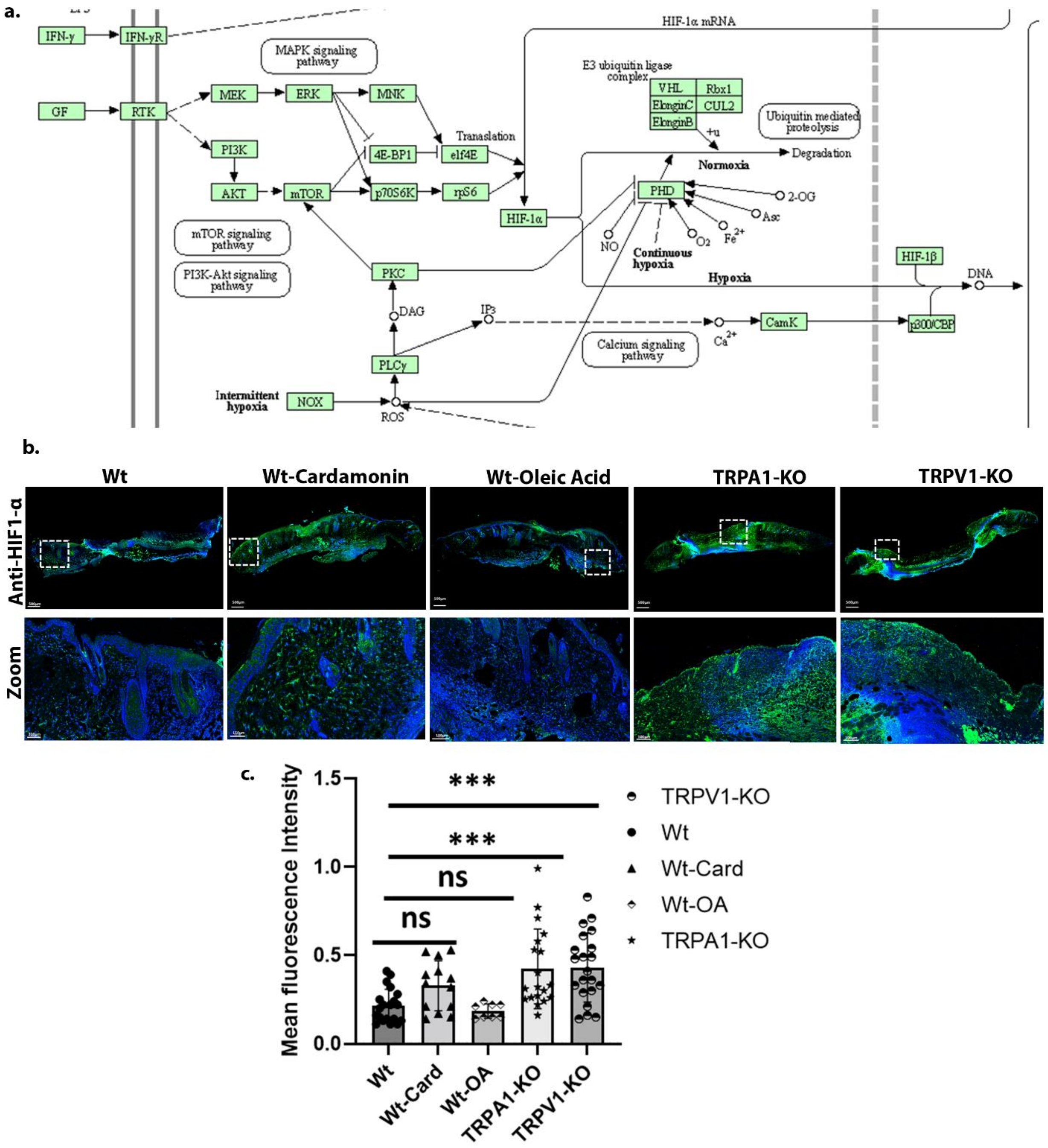

Supplementary Figure S2: String analysis reveals interesting pathways. (a). Impact on HIF-1a and MAPK pathways in TRPA1$^{-/-}$ mice by STRING analysis. **(b).** Confocal images of MRSA infected tissue stained with HIF1α antibody. Upper image shows whole tissue and lower images shows the enlarged view. **(c).** Fluorescence intensity quantification of HIF-1α stained images in each case. Graph pad prism software was used for statistical analysis. One way ANOVA (Dunnetts multiple comparisons test) was performed. The P-values are as follows *** <0.001, ** <0.01, * <0.05.

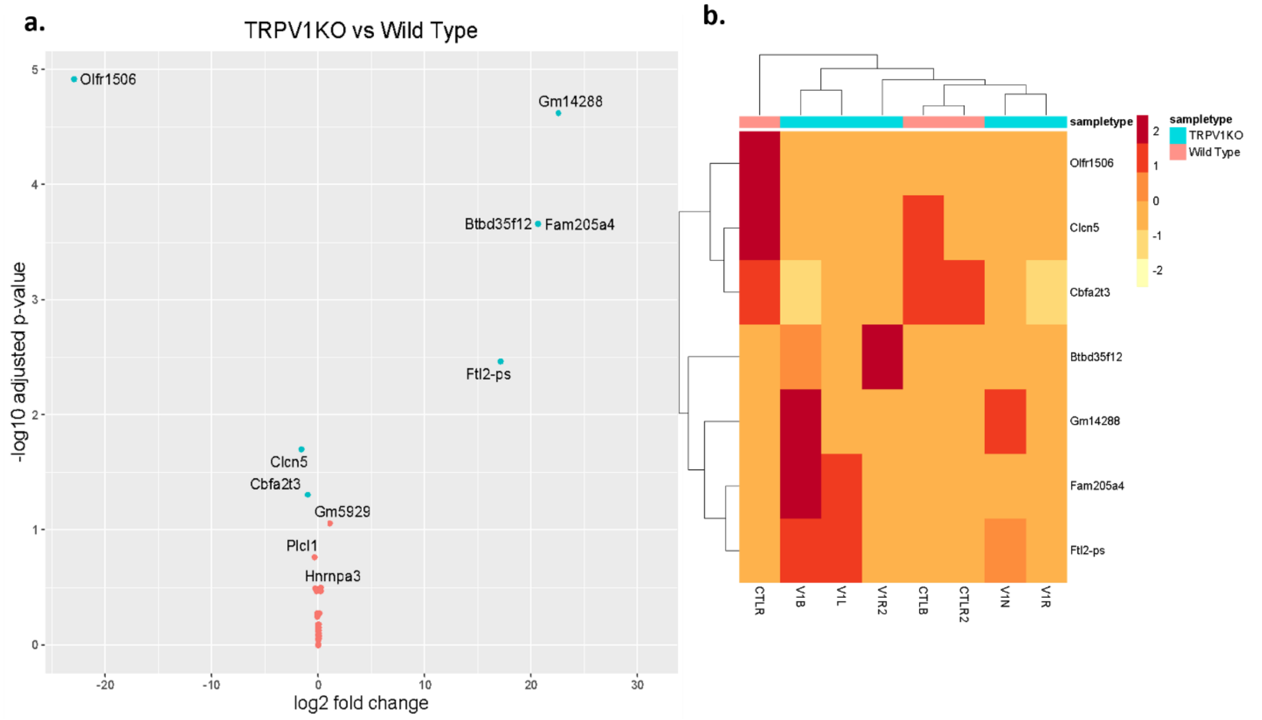

Supplementary Figure S3: Transcriptomics of TRPV1$^{-/-}$ mice tissue. (a). Volcano plot of differentially expressed genes (DEGs). Red dots represent non-significant genes, while green dots indicate significantly upregulated (fold change > 1.5) or downregulated (fold change < -1.5) genes. Threshold values for fold change are set at ±1.5. **(b).** Heatmap plot displays the patterns of differentially expressed genes (DEGs) across all sample types, highlighting sample to sample variations in each group.

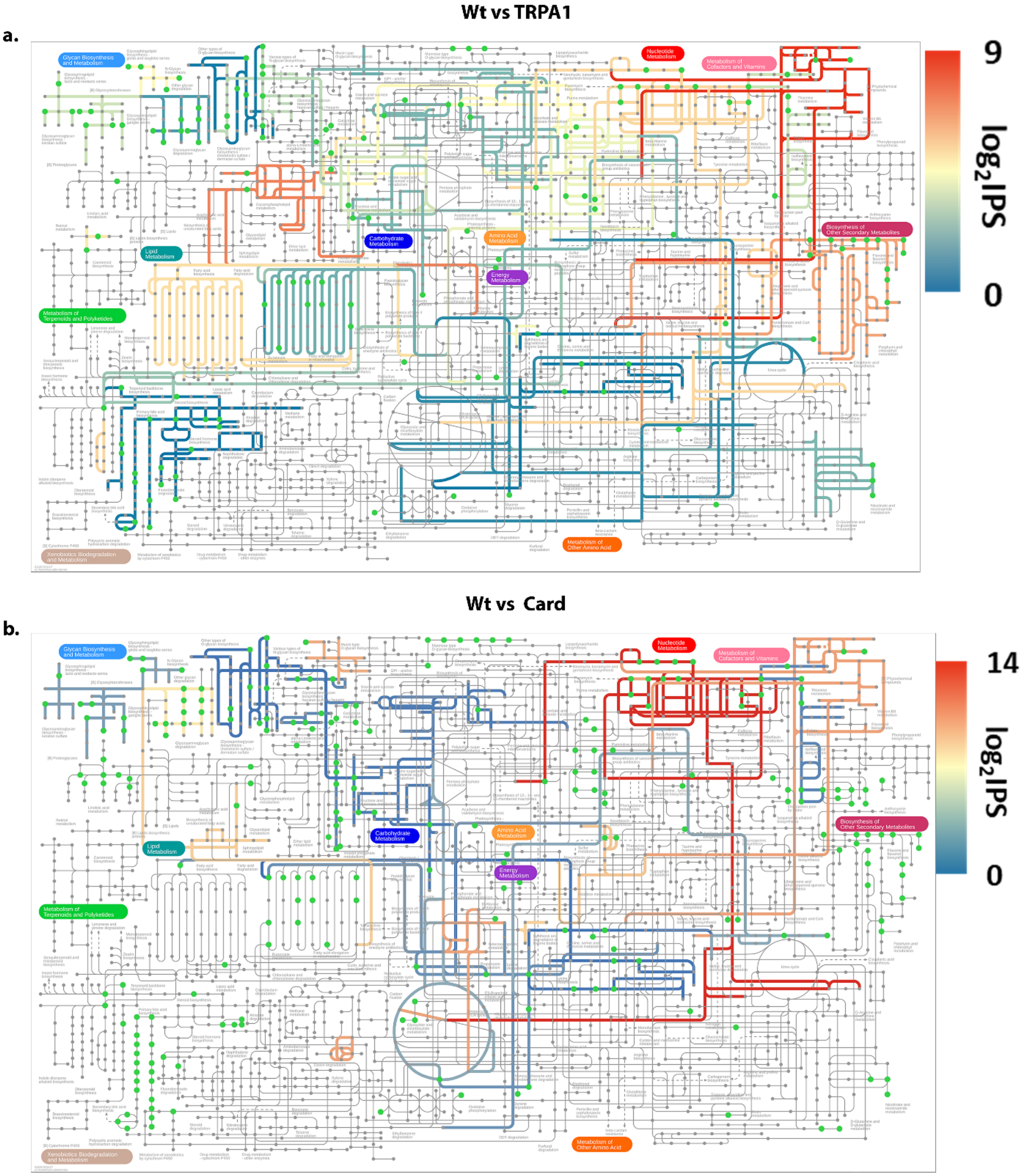

Supplementary Figure S4: Pathview map of significantly impacted pathways from the metabolomics data in TRPA1$^{-/-}$ mice. Pathway significance map showing enriched metabolic pathways in (TRPA1$^{-/-}$ or treated) versus controls. Index of Pathway Significance (IPS) was calculated from MetaboAnalyst outputs and-log scaled values were plotted on the KEGG map of metabolic pathways using iPath3. Pathways highlighted in red have high IPS values, blue have low IPS values, and gray pathways were not measured by our analysis. Green dots indicate chemical compounds matched to our dataset by MetaboAnalyst. **(a).** Shows pathview map from wild type vs TRPA1$^{-/-}$ pathways. **(b).** Shows pathview map from wild type vs cardamonin treated pathways.

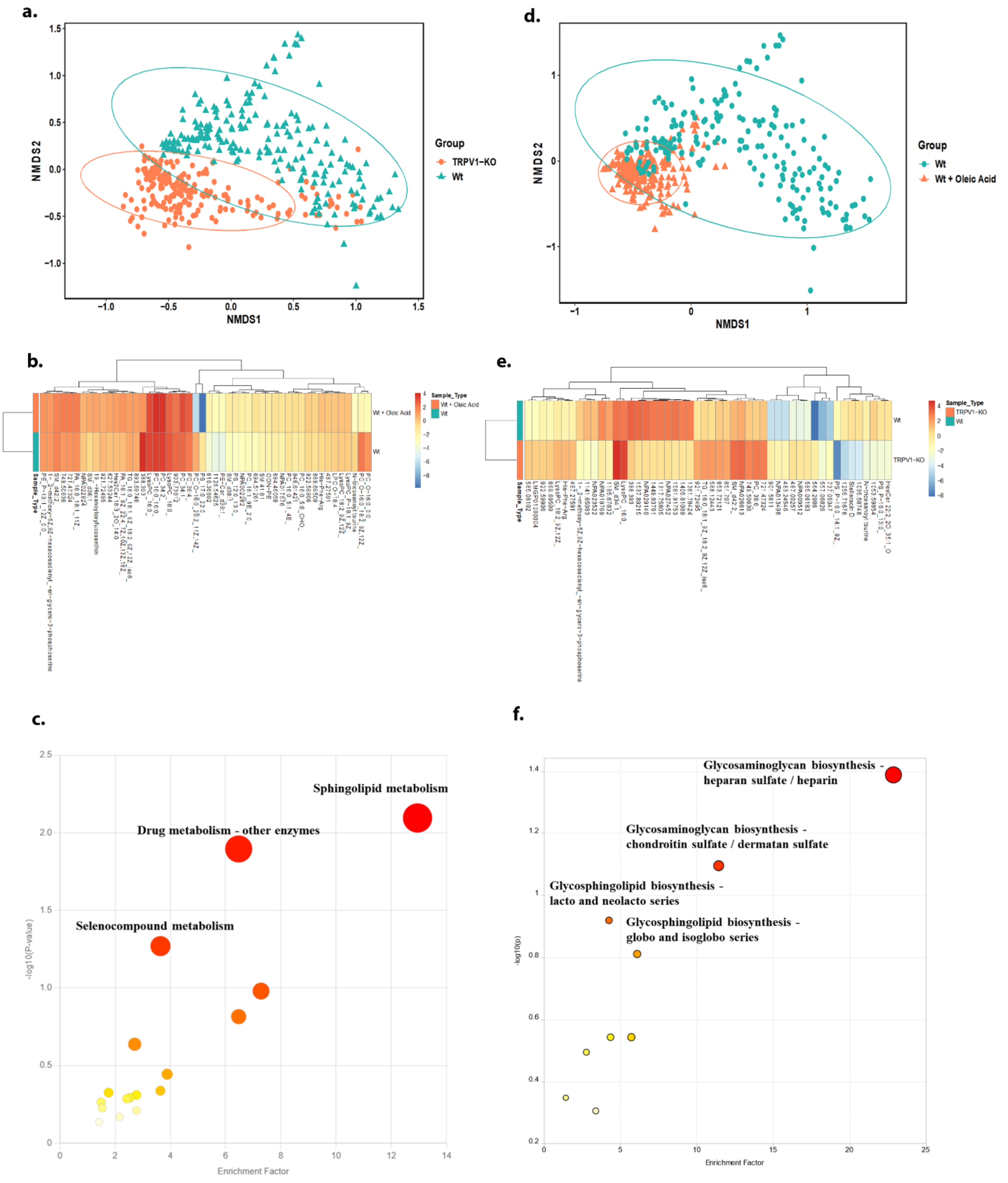

Supplementary Figure S5: Metabolomics of TRPV1$^{-/-}$ mice and oleic acid treated mice. (a). NMDS plot illustrates the differences in the overall metabolite profiles between wild type and oleic acid treated mice skin tissue. **(b).** Heat map plot shows the top 50 significant metabolites in wild type vs oleic acid treated mice. **(c).** Shows significantly impacted pathways hit from MetaboAnalyst in wild type and oleic acid treated mice skin tissue. **(d).** NMDS plot shows the differences in the total metabolites from wild type and TRPV1$^{-/-}$ treated mice skin tissue. **(e).** Heat map plot of significant

top 50 metabolites in wild type vs TRPV1$^{-/-}$ treated mice. **(f).** Significantly impacted pathways hit from MetaboAnalyst in wild type and TRPV1$^{-/-}$ treated mice skin tissue.

a.

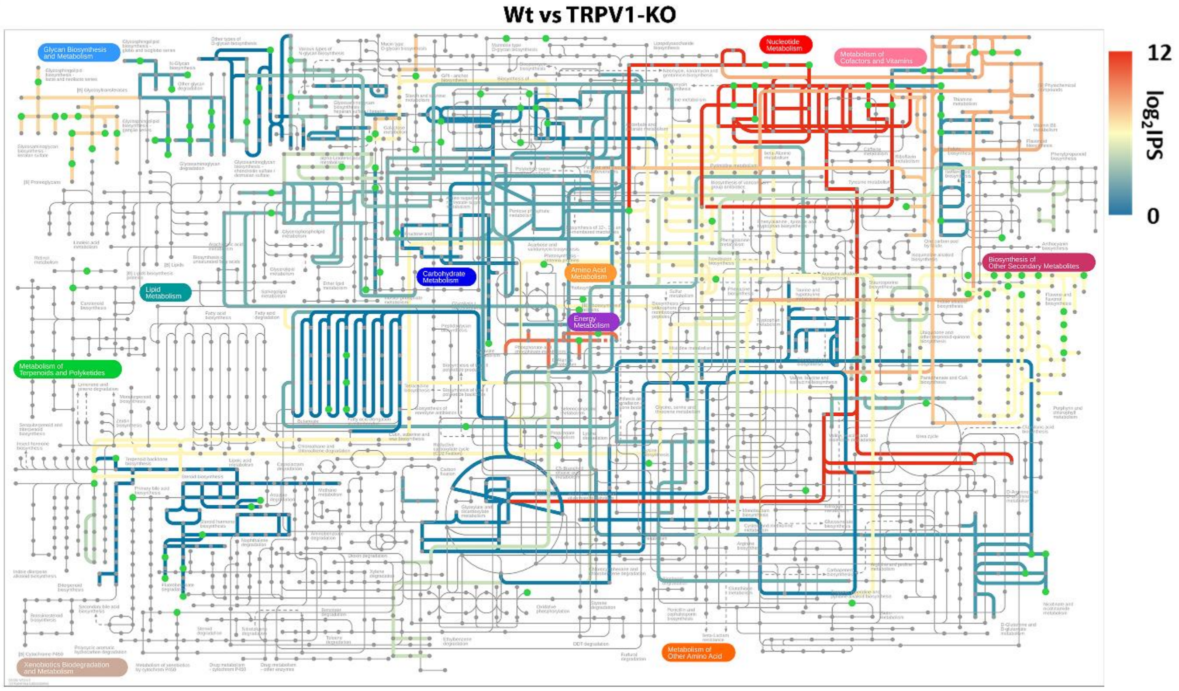

b.

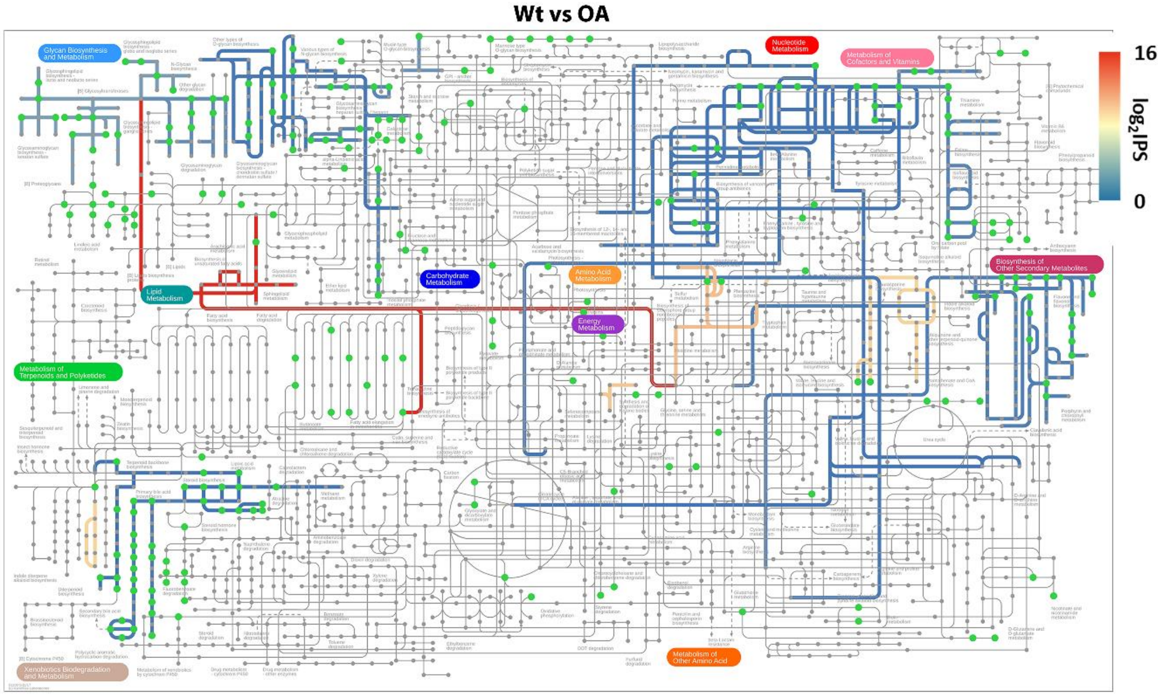

Supplementary Figure S6: Pathview map shows the significantly impacted pathways from the metabolomics data in TRPV1$^{-/-}$ mice. Pathway significance map showing enriched metabolic pathways in (TRPV1$^{-/-}$ or modulated) versus

controls. Index of Pathway Significance (IPS) was calculated from MetaboAnalyst outputs and-log scaled values were plotted on the KEGG map of metabolic pathways using iPath3. Pathways highlighted in red have high IPS values, blue have low IPS values, and gray pathways were not measured by our analysis. Green dots indicate chemical compounds matched to our dataset by MetaboAnalyst. **(a).** Pathview map from wild type vs TRPV1$^{-/-}$ pathways. **(b).** Pathview map from wild type and oleic acid treated pathways.

Supplementary Table S1: Integration of metabolomics and transcriptomics data reveals metabolites subclass which were found to be most impacted in the TRPA1$^{-/-}$ mice compared to wild type mice.

M/Z	NAME	CLASS	SUB CLASS
639.00326	ADP-D-ribose 1'',2''-cyclic phosphate	Purine nucleotides	Purine nucleotides sugars
707.39473	BMP 32:8	Glycerophosphoglycerols	Bismonoacylglycerophosphates)
829.95648	DG O-52:3	Diradylglycerols	O-DG (Alkyl,acylglycerols)
860.99495	Cer 18:1;O2/39:0	Ceramides	N-acylsphingosines (ceramides)
868.96171	Cer 14:0;O2/44:4	Ceramides	DHCer (Dihydroceramides)
925.01118	Cer 18:0;O2/44:4	Ceramides	DHCer (Dihydroceramides)
933.96395	Cer 22:1;O2/39:0	Sphingolipids	Ceramides
935.58511	SMGDG O-41:5	Glycosyldiradylglycerols	O-SMGDG (Semino lipids)
983.64486	SMGDG O-45:6;O	Glycosyldiradylglycerols	O-SMGDG (Semino lipids)
1010.08631	FAHFA 68:3;O	Fatty esters	FAHFA (Fatty acid ester of hydroxy fatty acids)
1015.00925	Cer 25:0;O2/42:2;O	Ceramides	DHCer (Dihydroceramides)
1023.64046	DGDG O-44:12	Glycosyldiradylglycerols	O-DGDG
1044.96834	DGTS 56:2	Other Glycerolipids	DGTS (Diacylglyceryl trimethylhomoserines)
1067.58786	PIM1 40:9	Glycerophosphoinositolglycans	PIM1 (Phosphatidylinositol monomannosides)
1111.66764	MLCL 49:9	Glycerophosphoglycerophosphoglycerols	Monolysocardiolipins)
1243.00856	SMGDG O-63:3	Glycosyldiradylglycerols	O-SMGDG (Semino lipids)
1417.95186	CL 66:1	Glycerophospholipids	Cardiolipins
1505.996	CL 74:5	Glycerophospholipids	Cardiolipins
1561.95434	CL 78:13	Glycerophospholipids	CL (Cardiolipins)
1572.99017	CL 80:11	Glycerophospholipids	CL (Cardiolipins)
1391.06291	Hex2Cer 73:10;O2	Glycosphingolipids	Hex2Cer
674.94453	Not annotated		
755.02824	Not annotated		
770.98506	Not annotated		
	AHexCer _O-26:6_18:1_2O_24:4_O	Other Glycerolipids	DGCC (Diacylglyceryl-3-O-carboxyhydroxymethylcholines)
	22-dimethylarsinoyl-_5Z,8Z, 11Z,14Z,17Z,20Z_-docosahexaenoic acid	Fatty Acyls [FA]	Other Fatty Acyls [FA00]
	TG_20:1_11Z_22:1_13Z_22:3_10Z,13Z,16Z_iso6_	PC(20:1(11Z)/22:1(13Z))	Glycerophospholipids [GP]
	NPA019456	not identified	
